# ROS-Induced Mitochondrial Dysfunction in CD4 T Cells from ART-Controlled People Living with HIV

**DOI:** 10.3390/v15051061

**Published:** 2023-04-26

**Authors:** Madison Schank, Juan Zhao, Ling Wang, Lam Ngoc Thao Nguyen, Yi Zhang, Xiao Y. Wu, Jinyu Zhang, Yong Jiang, Shunbin Ning, Mohamed El Gazzar, Jonathan P. Moorman, Zhi Q. Yao

**Affiliations:** 1 Center of Excellence in Inflammation, Infectious Disease and Immunity, James H. Quillen College of Medicine, East Tennessee State University, Johnson City, TN 37614, USA; 2 Department of Internal Medicine, Division of Infectious, Inflammatory and Immunologic Diseases, Quillen College of Medicine, East Tennessee State University, Johnson City, TN 37614, USA; 3 Hepatitis (HCV/HBV/HIV) Program, James H. Quillen VA Medical Center, Department of Veterans Affairs, Johnson City, TN 37614, USA

**Keywords:** oxidative stress, mitochondrial dysfunction, T cell aging, PLWH

## Abstract

We have previously demonstrated mitochondrial dysfunction in aging CD4 T cells from antiretroviral therapy (ART)-controlled people living with HIV (PLWH). However, the underlying mechanisms by which CD4 T cells develop mitochondrial dysfunction in PLWH remain unclear. In this study, we sought to elucidate the mechanism(s) of CD4 T cell mitochondrial compromise in ART-controlled PLWH. We first assessed the levels of reactive oxygen species (ROS), and we observed significantly increased cellular and mitochondrial ROS levels in CD4 T cells from PLWH compared to healthy subjects (HS). Furthermore, we observed a significant reduction in the levels of proteins responsible for antioxidant defense (superoxide dismutase 1, SOD1) and ROS-mediated DNA damage repair (apurinic/apyrimidinic endonuclease 1, APE1) in CD4 T cells from PLWH. Importantly, CRISPR/Cas9-mediated knockdown of SOD1 or APE1 in CD4 T cells from HS confirmed their roles in maintaining normal mitochondrial respiration via a p53-mediated pathway. Reconstitution of SOD1 or APE1 in CD4 T cells from PLWH successfully rescued mitochondrial function as evidenced by Seahorse analysis. These results indicate that ROS induces mitochondrial dysfunction, leading to premature T cell aging via dysregulation of SOD1 and APE1 during latent HIV infection.

## 1. Introduction

Human Immunodeficiency Virus (HIV) remains a major global health concern, with approximately 38.4 million individuals chronically infected with HIV, despite advancements in antiretroviral therapy (ART) for people living with HIV (PLWH). Current ART can significantly suppress HIV replication but cannot eliminate the integrated proviral DNA in reservoir cells. Thus, PLWH still present increased incidences of comorbidities and dampened immune responses [1,2,3]. Despite virologic control by ART, the hallmarks of latent HIV infection are failed reconstitution of memory CD4 T-cell responses, including those against HIV-1, immune overactivation, exhaustion, senescence, and persistent inflammation, along with increased oxidative stress. Latent HIV infection induces genomic instability, DNA damage, telomere erosion, and mitochondrial dysfunction, ultimately resulting in premature CD4 T cell aging in PLWH [1,2,3,4,5,6,7,8,9,10,11,12,13,14,15,16,17,18,19,20,21].

We have previously shown an accelerated loss of telomeres in CD4 T cells infected by HIV-1 in vitro and from PLWH, suggesting excessive proliferative turnover, telomeric DNA damage, and failure of DNA repair machinery [15,16,18,22]. We have also demonstrated increased CD4 T cell exhaustion and senescence and oxidative stress-mediated mitochondrial dysfunction during chronic hepatitis C virus (HCV) and HIV infections [15,16,17,18,19,23,24,25,26,27,28]. Mitochondrial dysfunction is a hallmark of cell aging and is commonly observed in high-turnover tissues such as T lymphocytes [29,30]. The basic functions of mitochondria (including cellular respiration) primarily rely on the transcription of mitochondrial DNA (mtDNA) [31,32,33]. Several critical processes are involved in mtDNA homeostasis and maintenance, including mtDNA replication, mitophagy, mitochondrial fusion and fission, and heteroplasmy, implicating a delicate balance between mtDNA damage and repair [34]. 

Reactive oxygen species (ROS) are produced during oxidative phosphorylation (OXPHOS) and function as important cellular signaling molecules. However, over-production of ROS can also induce mtDNA damage that is linked to mitochondrial compromise in HIV infection, as well as in cancer or neurodegenerative and cardiovascular diseases [35,36,37]. Given the accelerated rates of premature aging and elevated oxidative stress during HIV infection, PLWH may experience abrupt disruption to mtDNA. Recently, research has highlighted the connection between excessive ROS production, mitochondrial dysfunction, mtDNA damage, and cell aging [14,28,38,39]. We recently uncovered a crosstalk pathway by which targeted ROS production at mitochondria or telomeres induces telomeric DNA damage and mitochondrial dysfunction, respectively, in human T cells [38]. Thus, it is highly likely that an intrinsic mechanism exists by which HIV infection induces increased oxidative stress, which promotes DNA damage at both the nuclear and mitochondrial levels. The resulting deregulated cellular respiration and ATP production further amplifies DNA damage and accelerates aging in PLWH. The questions remaining unanswered are whether restoring ROS and/or mtDNA repair can rescue mtDNA levels and mitochondrial functions. We have previously shown that ROS are elevated in CD4 T cells from ART-controlled PLWH, and that mtDNA is enriched for 8-oxoguanine (8-oxoG), a hallmark of ROS-mediated DNA damage [28]. Because our study subjects are all stably HIV-suppressed by ART, we thus hypothesized that ROS-mediated impairment of mtDNA is a major cause of mitochondrial dysfunction in ART-controlled, virus-suppressed, latent HIV infection.

In order to maintain a fine-tuned balance in intracellular ROS production, cells express antioxidant defense molecules, such as superoxide dismutase 1 (SOD1), which catalyzes the dismutation of superoxide radicals. An essential role of SOD1 has been implicated across various disease states, including amyotrophic lateral sclerosis (ALS), motor neuropathy, cancer, and inflammatory diseases [37,40,41,42]. SOD1 primarily localizes to the cytosol but also localizes to the nucleus and the mitochondrial intermembrane space (IMS), matrix, and outer membrane. SOD1 enzymatic activity requires a series of posttranslational, stabilizing modifications that occur once SOD1 protein is transported to mitochondria, trapping SOD1 in the IMS [37,43,44,45,46]. SOD1 mutation in mice significantly reduces electron transport, mitochondrial respiration, and ATP production while increasing oxidative damage to mitochondrial proteins [44]. SOD1 has also been shown to associate with mitochondria in a tissue- and age-dependent manner in rodents. Notably, loss of SOD1 induces senescence in human fibroblasts [37,41]. 

Furthermore, cells express DNA damage repair proteins, including apurinic/apyrimidinic endonuclease 1 (APE1), which is able to rescue DNA damage resulting from elevated oxidative stress. APE1 is a multifunctional protein with both nuclease and transcriptional regulatory activities. APE1 regulates cellular DNA repair and redox signaling via the base excision repair (BER) pathway. BER is the major DNA repair pathway that is active in mitochondria. Nevertheless, DNA damage repair pathways and antioxidant protection become disrupted with advanced aging [36,47]. APE1 has a mitochondrial-targeted localization signal and has been shown to reduce oxidative stress upon mitochondrial localization. A recent study has also shown post-translational modification via arginine methylation of APE1, which triggers APE1 translocation to the mitochondria and increases protection from oxidative DNA damage [36]. Notably, in osteosarcoma cell lines, APE1 knockdown (KD) increased ROS and DNA damage, while overexpression (OE) of APE1 reduced cell apoptosis [48]. These studies highlight the essential role of SOD1 and APE1 in maintaining normal mitochondrial and cellular functions. 

In this study, we employed a translational approach by using CD4 T cells from PLWH and healthy subjects (HS) to determine the role of oxidative stress and deregulation of critical molecules involved in mitochondrial compromise to accelerate CD4 T cell aging in PLWH. We demonstrated increased cellular and mitochondrial ROS in CD4 T cells from PLWH. Using Liquid Chromatography Mass Spectrometry (LC-MS), we evaluated the expression of critical antioxidant and DNA damage repair mitochondrial proteins and observed significantly reduced levels of SOD1 and APE1. Knockdown of SOD1 or APE1 in HS significantly impaired mitochondrial respiration likely via p53 upregulation, and overexpression of these proteins rescued cellular respiration in CD4 T cells from PLWH. Taken together, these results suggest an important role of SOD1 and APE1 and highlight a multi-layer mechanism influencing mitochondrial dysfunction during latent HIV infection. 

## 2. Materials and Methods

### 2.1. Subjects

The study subjects included two populations: blood samples from 77 PLWH on ART with undetectable viremia (HIV-RNA < 20 copies/mL) and 46 age-matched HS (obtained from BioIVT, Gray, TN, USA) who were all negative for HBV (hepatitis B virus), HCV, and HIV infections. All subjects gave their informed consent for inclusion before participation in the study. This study was performed in accordance with the Declaration of Helsinki and the joint institutional review board (IRB) of East Tennessee State University (ETSU) and James H. Quillen VA Medical Center (Johnson City, TN, USA). 

### 2.2. Cell Isolation and Culture

PBMCs were isolated from whole blood using Ficoll density centrifugation (GE Healthcare; Piscataway, NJ, USA). CD4 T cells were isolated from PBMCs using a CD4 T Cell Negative Selection Kit (Miltenyi; Cambridge, MA, USA). CD4 T cells were cultured in RPMI-1640 medium containing 10% fetal bovine serum (FBS) (Atlanta Biologicals, Flowery Branch, GA, USA), 100 IU/mL penicillin, and 2 mM L-glutamine (Thermo Scientific, Logan, UT, USA), and maintained at 37 °C in a 5% CO_2_ incubator.

### 2.3. Flow Cytometry

Cellular ROS in CD4 T cells was detected using the CellROX Green ROS Detection kit according to the manufacturer’s protocol (Thermo Fisher Scientific; Waltham, MA, USA). Mitochondrial ROS was assessed by staining cells with 5 μM MitoSOX Red mitochondrial superoxide indicator (Invitrogen; Waltham, MA, USA) at 37 °C for 30 min, washed 3 times, and then analyzed using flow cytometry. For mitochondrial function analysis, the MG (Cat #M-7514) and MO (Cat #M-7511) (Invitrogen) probes were used according to the manufacturer’s instructions. The MG and MO mitochondrion-selective probes allow for the quantification of mitochondrial mass and oxidative phosphorylation (OXPHOS), respectively. The cells were cultured with 100 nM of MG or 500 nM of MO for 30 min at 37 °C. For apoptosis analysis, the cells were washed twice with DPBS and stained with Annexin V-PE (Cat #BDB556422) (BD Biosciences; San Jose, CA, USA) in 1X binding buffer for 15 min according to the manufacturer’s protocol. Controls for these assays included unstained cells, isotype control antibodies, and single positive staining, which were used for gating and compensation. Samples were analyzed using a BD AccuriC6 Plus flow cytometer and FlowJo V10 software.

### 2.4. Mitochondrial Purification

Mitochondrial and cytosolic fractionation were performed using the Qproteome Mitochondria Isolation Kit from (QIAGEN; Germantown, MD, USA) according to the manufacturer’s protocol. Following fractionation, the cytosolic fraction was concentrated using an Amicon Ultra-0.5 Centrifugal 10K Filter Unit (Millipore-Sigma; Saint Louis, MO, USA) according to the manufacturer’s instructions. The mitochondrial pellet was lysed on ice in RIPA lysis buffer (Boston BioProducts; Ashland, MA, USA) in the presence of protease inhibitors (Thermo Fisher Scientific).

### 2.5. Liquid Chromatography-Tandem Mass Spectrometry (LC-MS/MS)

LC-MS was performed at the Center for Integrative Proteomics Research of the Robert Wood Johnson Medical School and Rutgers University, using a method as previously reported [49,50]. Mitochondria were fractionated from CD4 T cells isolated from 3 HS and 3 PLWH. Approximately 5 µg mitochondrial proteins were assessed using an LC-MS shotgun approach at the Center for Integrative Proteomics Research of the Robert Wood Johnson Medical School and Rutgers University following their standard protocol. Label-free quantification (LFQ) was used to determine the relative protein amounts as a fold change [49]. 

### 2.6. Western Blotting

CD4 T cells were lysed on ice in RIPA lysis buffer (Boston BioProducts) in the presence of protease inhibitors (Thermo Fisher Scientific). Protein concentration was determined using the Pierce BCA protein assay kit (Thermo Fisher Scientific). Proteins were separated using SDS-PAGE and transferred to polyvinylidene difluoride membranes. The membranes were blocked with 5% non-fat milk, 0.1% Tween-20 in Tris-buffered saline (TBS) and incubated overnight with primary antibodies for the indicated proteins. Primary antibodies were obtained from Cell Signaling Technology (Danvers, MA, USA), Thermo Fisher Scientific, or Santa Cruz Biotechnology (Dallas, TX, USA). Appropriate horseradish peroxide-conjugated secondary antibodies (Cell Signaling) were added for 1 h at room temperature, and the proteins were visualized using the Amersham ECL Prime Western Blotting Detection Reagent (GE Healthcare Bio-Sciences; Pittsburgh, PA, USA). The protein bands were captured and quantified using the Chemi DocTM MP Imaging System (Bio-Rad; Hercules, CA, USA) and normalized to the β-Actin (Cell Signaling) loading control.

### 2.7. Reverse Transcription Quantitative PCR (RT-qPCR)

Total RNA was extracted from 2-day cultured CD4 T cells from PLWH or HS with the PureLink RNA Mini Kit (Invitrogen). cDNA was synthesized with the High-Capacity cDNA Reverse Transcription Kit (Applied Biosystems, Foster City, CA, USA) according to the manufacturer’s protocol. The qPCR was performed in triplicate. Primers included: SOD1 Forward 5′-ACTGGTGGTCCATGAAAAAGC-3′, Reverse 5′-AACGACTTCCAGCGTTTCCT-3′; APE1 Forward 5′-GTTTCTTACGGCATAGGCGAT-3′, Reverse 5′-CACAAACGAGTCAAATTCAGCC-3′; and GAPDH Forward 5′-GGATTTGGTCGTATTGGG-3′, Reverse 5′-GGAAGATGGTGATGGGATT-3′. The cycling conditions were 1 cycle at 95 °C for 3 min, followed by 40 cycles at 95 °C for 10 s and 60 °C for 30 s. Gene expression was calculated using the 2^−ΔΔct^ method, normalized to GAPDH levels, and is presented as fold change.

### 2.8. CRISPR/Cas9-Mediated Knockdown

CD4 T cells were purified from PBMCs of HS and stimulated with soluble anti-CD3 (2 μg/mL) and anti-CD28 (4 μg/mL) antibodies for 3 days in 10% FBS cRPMI with 30 IU/mL IL-2. The SOD1, APEX1, or Peroxiredoxin 1 (PRDX1) crRNP (crRNA and tracrRNA purchased from Dharmacon, Lafayette, CO, USA) was formed following a previously published protocol [51] and used to transfect the stimulated CD4 T cells using the Lonza P3 Primary Cell 4D X Kit L and program EH-115, following the manufacturer’s instructions (Cat #V4XP-3024, Lonza; Basel, Switzerland). The cells were harvested on day 3 after nucleofection for analysis.

### 2.9. Quantification of mtDNA Copy Number

Genomic DNA was extracted from CD4 T cells using the PureLink genomic DNA kit (Thermo Fisher Scientific). DNA concentration was measured with a Synergy H1 BioTek plate reader. The primers used for mitochondrial and nuclear DNA qPCR were: mtDNA tRNALeu Forward 5′-CACCCAAGAACAGGGTTTGT-3′, Reverse 5′-TGGCCATGGGTATGTTGTTA-3′; and nuDNA β2-microglobulin Forward 5′-TGCTGTCTCCATGTTTGATGTATCT-3′, Reverse 5′-TCTCTGCTCCCCACCTCTAAGT-3′. For mtDNA/nuDNA ratios, approximately 25 ng of genomic DNA were used in PCR reactions. The PCR cycling conditions were 1 cycle at 50 °C for 2 min, 1 cycle at 95 °C for 10 min, and 40 cycles at 95 °C for 15 s and 62 °C for 60 s. The averages of mtDNA and nuDNA Cq values from triplicate reactions were calculated and mitochondrial DNA content was determined using the following equation: a. ΔCq = (nuDNA Cq − mtDNA Cq) b. Relative mitochondrial DNA content = 2 × 2^ΔCq^ [52]. 

### 2.10. Quantification of mtDNA Damage

For quantification of the percentage of mtDNA damage, we used the Human real-time PCR mitochondrial DNA damage analysis kit (Detroit R&D, Cat #DD2H) according to the manufacturer’s instructions. This kit allows for the measurement of human 8.8-kb mtDNA in vitro by quantification of the replicated DNA with real-time qPCR. Genomic DNA was extracted from CD4 T cells using the PureLink genomic DNA kit. Briefly, 1 uL of 50 ng/uL DNA was combined with 10 uL of 2× QPCR concentrated buffer, 4 uL of 5× Enhancer, and 5 uL of QPCR primer mix (2 µM each primer, forward/reverse). Each PCR reaction was performed in duplicate. The qPCR cycling conditions were 1 cycle at 98 °C for 30 s, 30 cycles at 98 °C for 10 s, 68 °C for 10 s, and 72 °C for 4 min, followed by 72 °C for 10 min. The qPCR DNA product was diluted 10-fold with nuclease-free water and used for real-time qPCR. A standard dilution was made, ranging from 0 to 1 ng/uL. One microliter of diluted qPCR DNA product was combined with 10 uL of SYBR mix (Bio-Rad), 8.1 uL water, and 0.9 uL of real-time primer mix (5 µM each primer). The real-time qPCR cycling conditions were 1 cycle at 50 °C for 2 min, 95 °C for 10 min, followed by 40 cycles at 95 °C for 15 s and 60 °C for 60 s. A standard curve was made using the threshold cycle value (CT) and the DNA concentration (log scale) of each of the 8.8-kb standards. Linear regression analysis was used to calculate the DNA concentration of the samples and the percentage of mtDNA damage.

### 2.11. Seahorse Respiration Studies

Seahorse XFp Cell Mito Stress Tests (Seahorse; Agilent Technologies) were completed following the manufacturer’s protocol using an XFp instrument. Briefly, one day before the assay, Seahorse mini-cartridges were hydrated at 37 °C overnight in a non-CO_2_ incubator. On the day of the assay, seahorse mini plates were coated with 25 μL of 0.1 mg/mL poly-D-lysine (Cat #A3890401) (ThermoFisher Scientific) for 1 h at room temperature. CD4 T cells were harvested following nucleofection. Approximately, 2 × 10^5^ cells per well were plated into pre-coated mini plates and cultured in Seahorse XF RPMI-1640 medium supplemented with 1.0 mM Glucose, 100 µM Pyruvate, and 1.0 mM Glutamine. Data analysis were performed using the Seahorse Wave software.

### 2.12. Lentiviral Packaging and Overexpression

HEK293T cells were cultured to 80% confluency and then transfected with 5 μg of pMD2.G (Cat #12259), 5 μg of psPAX.2 (Cat #12260) (gift from Dr. Didier Trone; Addgene), and 10 μg of transfer vector pLenti-C-Myc-DDK (Cat# PS100064V), pLenti-C-Myc-DDK-SOD1 (Cat# RC200725L1), or pLenti-C-Myc-DDK-APEX1 (Cat# RC201732L1) (all from OriGene; Rockville, MD, USA) using the Transporter 5 Transfection reagent (PEI, Polysciences; Warrington, PA, USA). The lentiviruses were harvested, filtered with a 0.45 μm sterile syringe filter, and concentrated with the PEG-it virus precipitation solution (System Biosciences, Palo Alto, CA, USA) according to the manufacturer’s instructions. The lentiviruses were resuspended in sterile DPBS and stored at −80 °C. 

Purified CD4 T cells from PLWH were stimulated with anti-CD3/CD28 dynabeads (Invitrogen) at a ratio of 2 cells:1 bead for 24 h in RPMI supplemented with 10% FBS, with 30 IU/mL IL-2 (Cat #589104; BioLegend, San Diego, CA, USA). The cells were harvested, washed to remove anti-CD3/CD28 beads, and subjected to lentiviral transduction with the pLenti, pLenti-SOD1, or pLenti-APEX1 encoding viruses. The TransDux™ MAX Lentivirus Transduction Reagent (System Biosciences) was used to enhance transduction efficiency following the manufacturer’s protocol. Briefly, stimulated CD4T cells were resuspended with 400 mL RPMI complete medium, 100 μL of MAX Enhancer, 2 μL of TransDuxTM, and 4 μL of 1M HEPES buffer. Approximately 2.5 × 10^6^ cells were then seeded into a 24-well plate with the appropriate lentivirus and then centrifuged at 1500× *g* at 32 °C for 2 h. The supernatant was discarded and cells were resuspended with 2 mL RPMI complete media supplemented with 1 µg/mL anti-CD3 and 2 µg/mL anti-CD28 antibodies and 30 IU/mL IL-2. The cells were collected at 48 h post-transduction and subjected to downstream experiments.

### 2.13. Statistical Analysis

The data were analyzed using Prism 9 software and are presented as mean ± SEM. The outliers were identified using the ROUT method (Q = 1.000%) and excluded from the analysis. Comparisons between two groups for normal distribution were made using a parametric unpaired *t*-test (equal SD) or parametric unpaired *t*-test with Welch’s correction (unequal SD) and then analyzed using Pearson correlation. Comparisons between two groups for skewed distribution data were made using the non-parametric Mann–Whitney U test. Comparisons between paired groups were made using a parametric paired *t*-test for normally distributed data or a non-parametric Wilcoxon paired *t*-test for non-normal distributions. The *p*-values of <0.05 were considered statistically significant.

## 3. Results

### 3.1. Cellular and Mitochondrial ROS Are Increased in CD4 T Cells from PLWH

**Figure 1:**
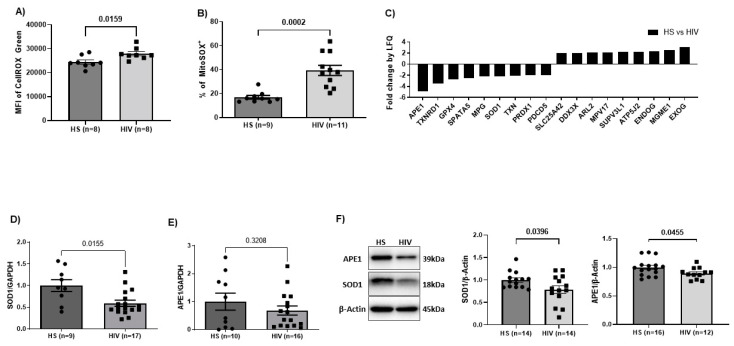
ROS production and SOD1/APE1 protein expression in CD4 T cells of PLWH and HS. (**A**,**B**) The MFI of CellROX Green (cellular ROS) or the frequency of MitoSOX+ (mitochondrial ROS) in CD4 T cells from PLWH or HS following 2-day culture. (**C**) LC-MS identification and label-free quantification (LFQ) of proteins isolated from mitochondria of HS (*n* = 3) and ART-treated PLWH (*n* = 3). Mitochondrial proteins were assessed using an LC-MS shotgun approach. CD4 T cells from 3 HS or PLWH were pooled to get enough mitochondria for this analysis. The data are a subset of proteins that are known to play a critical role in mtDNA maintenance and have >2-fold change in HIV samples versus HS, as determined by a standard LFQ method. (**D**,**E**) RT-qPCR analysis of mRNA expression level of SOD1 or APE1 in CD4 T cells from PLWH or HS following 2-day culture. mRNA expression of SOD1 or APE1 was normalized to GAPDH. (**F**) Representative and summary data for Western blot analysis of protein levels of SOD1 or APE1 in CD4 T cells from HS or PLWH normalized to β-Actin.

Oxidative stress is a prominent feature of dysfunctional CD4 T cells during chronic viral infections [19,24,53]. To investigate the underlying mechanism of CD4 T cell dysfunction during latent HIV infection, we first measured the levels of cellular and mitochondrial ROS in CD4 T cells from PLWH and HS. The demographic features of the subjects used in this study are shown in Table 1. Additional clinical information is provided in Supplementary Tables S1 and S2. We observed significantly higher levels of cellular ROS, as determined by the MFI of CellROX Green (Figure 1A), and mitochondrial ROS, as measured by the percentage of MitoSOX^+^ (Figure 1B) and MFI of MitoSOX (data not shown), in CD4 T cells from PLWH versus HS, indicating that CD4 T cells undergo increased oxidative stress during latent HIV infection. We have previously shown that CD4 T cells from PLWH have a significantly reduced mtDNA content compared to HS and significantly increased 8-oxoG in mtDNA [28]. Furthermore, we have demonstrated increased topoisomerase 1 cleavage complex (Top1cc), indicating failure to reverse DNA entanglement for normal repair, and cleaved poly [ADP-ribose] polymerase 1 (PARP1) in mitochondria of CD4 T cells from chronically infected HCV and HIV individuals [54]. These data indicate that following HIV infection, mtDNA becomes oxidatively damaged and may experience compromised mtDNA repair and thus increased degradation. We have also shown impaired CD4 T cell respiration and ATP production during latent HIV infection [28]. Collectively, these data implicate ROS in inducing mtDNA damage, which impairs CD4 T cell mitochondrial functions.

### 3.2. Deregulation of Antioxidant and DNA Damage Repair Enzymes in CD4 T Cells from PLWH

To uncover the mechanisms of elevated oxidative stress, mtDNA reduction, and mitochondrial dysfunctions in PLWH, we assessed the protein composition of mitochondria isolated from CD4 T cells of ART-controlled PLWH using LC-MS. We screened 1023 known mitochondrial proteins and observed 148 proteins with >2-fold changes between ART-controlled PLWH and HS. Based on protein function descriptions in the Human Gene Database and Uniport, we selected enzymatic proteins that are known to have direct or indirect roles in regulating mtDNA functions, including nucleotide synthesis, mtDNA replication, mtDNA damage and repair signaling, mtDNA recombination, and mitophagy [33,55,56,57,58,59]. This approach yielded 18 mitochondrial proteins of interest, as shown in Figure 1C. The proteomic data showed a reduction in mitochondrial SOD1, which supports our finding that CD4 T cells from PLWH overproduce ROS. In addition, the decrease in APE1 in the mitochondrial fraction and the elevated 8-oxoG in mtDNA of HIV-CD4 T cells [28] suggest a significant defect in the BER pathway for DNA damage response (DDR), which we have previously demonstrated occurs with selectively oxidative stress in telomere targeting [27]. We further assessed the gene expression and total protein levels of SOD1 and APE1 in CD4 T cells. We observed a significant reduction in the mRNA level of SOD1 but not APE1 in CD4 T cells from PLWH compared to HS (Figure 1D,E). We did, however, find a significant reduction in both SOD1 and APE1 protein levels in CD4 T cells from PLWH versus HS (Figure 1F). These results demonstrate that the protein levels of SOD1 and APE1 are regulated at the transcriptional and post-transcriptional levels, respectively, in CD4 T cells during latent HIV infections, resulting in ROS-mediated oxidative mtDNA damage/reduction and mitochondrial dysfunction.

### 3.3. SOD1 Regulates CD4 T Cell Mitochondrial Respiration

**Figure 2:**
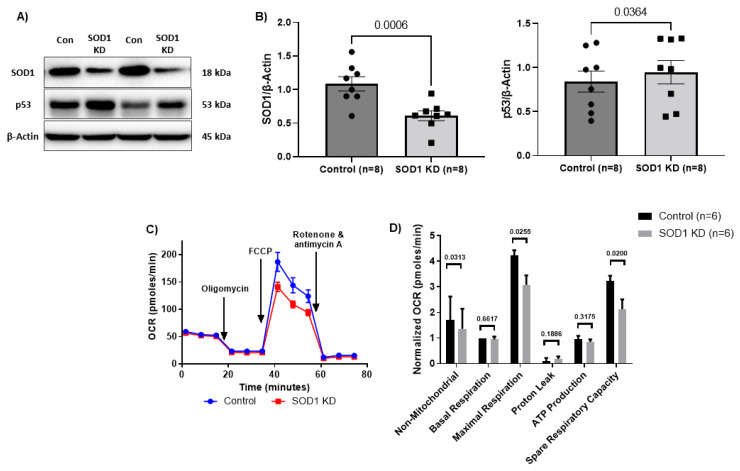
Mitochondrial functions following SOD1 knockdown in CD4 T cells from HS. (**A**,**B**) Representative Western blots and summary data of SOD1 and p53 protein levels following SOD1 KD in CD4 T cells from HS, normalized to β-Actin (*n* = 8). (**C**,**D**) Oxygen consumption rate (OCR) as measured using Seahorse XFp Cell Mito Stress Tests, and summary data for non-mitochondrial, basal, and maximal respiration, proton leak, ATP production, and spare respiratory capacity following SOD1 KD (*n* = 6). KD—knockdown.

SOD1 is a critical antioxidant molecule that regulates cellular oxidative stress. To determine the role of SOD1 in ROS-mediated mtDNA damage and mitochondrial dysfunction, we employed a CRISPR/Cas9 technique to knockdown (KD) SOD1 in CD4 T cells from HS. We synthesized target crRNA/tracrRNA with Cas9-NLS protein and transfected TCR-activated (for 72 h) CD4 T cells from HS. As shown in Figure 2A,B, an efficient SOD1 KD was observed at 72 h after transfection, as evidenced by a significant decrease in SOD1 protein levels compared with the control. Given the critical role of SOD1 in antioxidant metabolism, we next sought to evaluate the effect of this KD on the levels of oxidative stress, mtDNA copy number, mtDNA damage, cellular respiration, ATP production, and mitochondrial fitness. Surprisingly, we observed no significant changes in cellular ROS (Supplementary Figure S1A), mitochondrial ROS (Supplementary Figure S1B), Mito-Tracker Green (MG, a marker for mitochondrial mass, Supplementary Figure S1C), MitoTracker Orange (MO, a marker for mitochondrial membrane potential, Supplementary Figure S1D), mtDNA copy number (Supplementary Figure S1E), or percentage of mtDNA damage (Supplementary Figure S1F). This is likely due to the presence of multiple antioxidant signaling molecules functioning synergistically to protect from cellular oxidative stress.

We then utilized Seahorse Cell MitoStress tests to evaluate basal respiration, ATP production, proton leak, maximal respiration, spare respiratory capacity, and non-mitochondrial respiration in the treated cells. As shown in Figure 2C,D, we observed significantly reduced non-mitochondrial and maximal respirations, as well as spare respiratory capacity in SOD1-KD CD4 T cells from HS. Maximal respiration is energy demanding as the respiratory chain is stimulated to operate at maximum capacity, leading to the rapid oxidation of substrates (fats, sugars, and amino acids) to meet the metabolic requirements of the cell. Spare respiratory capacity represents the capability of the cell to respond to an energetic demand as well as how closely the cell is respiring to its theoretical maximum and thus is used as an indicator of cell mitochondrial fitness. The perturbations in maximal respiration and spare respiratory capacity indicate that SOD1-KD induces mitochondrial dysfunction. 

Given the alterations in mitochondrial functions following SOD1-KD but no significant changes in mtDNA copy number or percentage of mtDNA damage, we hypothesized that these functional changes may result from additional alterations induced by SOD1-KD independently of mtDNA. A previous study has shown that SOD1 deficiency induces DNA damage and cellular dysfunction by upregulating the critical DNA damage tumor protein p53 (p53) [60]. Thus, we evaluated the level of p53 following SOD1-KD. We observed a significant upregulation of p53 protein levels following SOD1-KD in HS-CD4 T cells (Figure 2A,B). Interestingly, we have previously shown that p53 is involved in the regulation of the master mitochondrial regulators, including peroxisome proliferator-activated receptor gamma coactivator 1 alpha (PGC-1α), estrogen-related receptor alpha (ERRα), nuclear respiratory factor (NRF-1), and mitochondrial transcription factor A (mtTFA) during telomere shortening and premature T cell aging and p53-KD rescued mitochondrial functions [27]. These findings highlight the role of SOD1 in maintaining mitochondrial functions by preventing DNA damage-mediated p53 activation.

**Figure 3:**
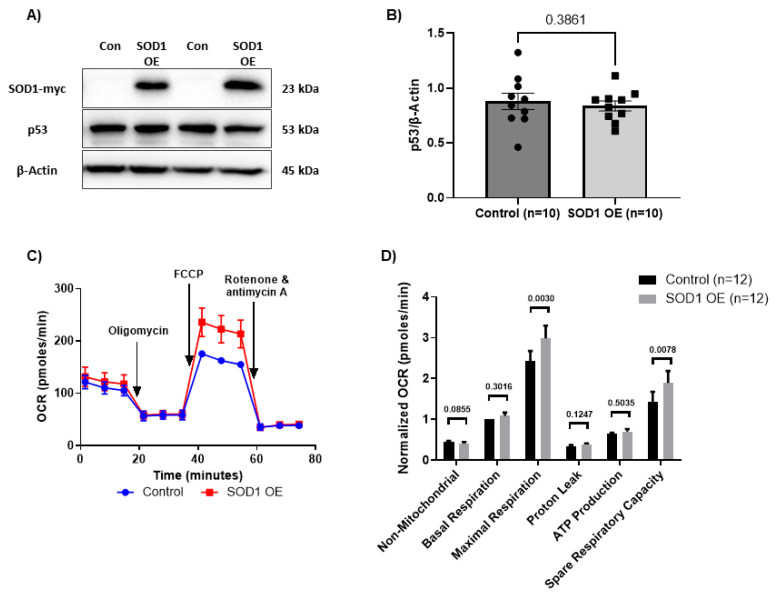
Mitochondrial functions following SOD1 overexpression in CD4 T cells from PLWH. (**A**,**B**) Representative Western blots and summary data of SOD1-myc and p53 protein levels following SOD1 OE in CD4 T cells from PLWH normalized to β-Actin (*n* =10). (**C**,**D**) OCR and summary data for non-mitochondrial, basal, and maximal respiration, proton leak, ATP production, and spare respiratory capacity following SOD1 OE (*n* = 12). OE—overexpression.

We next overexpressed SOD1 in HIV-CD4 T cells to determine if restoring SOD1 levels could rescue the cell mitochondrial functions. We observed successful OE of SOD1 as shown by the presence of the myc-SOD1 tagged protein following lentiviral transfection (Figure 3A). Again, we did not observe alterations in cellular ROS (Supplementary Figure S1G), mitochondrial ROS (Supplementary Figure S1H), MG (Supplementary Figure S1I), MO (Supplementary Figure S1J), and mtDNA copy number (Supplementary Figure S1K). However, we did observe a significant restoration of maximal respiration and spare respiratory capacity following SOD1-OE (Figure 3C,D). We further evaluated the level of p53 following SOD1-OE. Despite successful SOD1-OE, we did not observe a statistically significant change in the p53 protein levels (Figure 3B), likely due to multiple p53-regulatory factors existing in HIV-CD4 T cells. Taken together, these data indicate that deregulation of SOD1 does not directly alter mtDNA content and mitochondrial oxidative parameters, such as ROS levels, mitochondrial mass, or mitochondrial membrane potential, but is sufficient to inhibit (via KD in HS) or restore (via OE in HIV) cellular respiration and mitochondrial function in CD4 T cells, likely through a p53-dependent mechanism. 

The above described findings led us to investigate the effect of an additional antioxidant enzyme (Peroxiredoxin 1-PRDX1), as LC-MS analysis showed that PRDX1 is downregulated in mitochondria from CD4 T cells of PLWH, by performing PRDX1 KD in CD4 T cells from HS. We observed an efficient KD of PRDX1 (Supplementary Figure S2A). However, despite the efficient KD of PRDX1, we did not observe significant changes in the mtDNA content, cellular or mitochondrial ROS levels, MFI of MG, frequency of MO^+^, or cellular respiration (Supplementary Figure S2B–G). Thus, we did not assess alterations in mitochondrial functions following PRDX1-OE in CD4 T cells from PLWH. We attempted to evaluate the effect of dual knockdown of multiple antioxidant enzymes (e.g., simultaneous KD of SOD1 and PRDX1) on mitochondrial readouts, including mtDNA copy number and cellular and mitochondrial ROS. We did not find any significant alterations in these readouts following KD compared to the control (data not shown). Nevertheless, these data suggest that while SOD1-KD and SOD1-OE were not sufficient to alter mtDNA copy number or other mitochondrial readouts, these manipulations significantly influenced cellular respiration and mitochondrial function, but PRDX1-KD did not lead to such an effect. Therefore, this suggests a critical role of SOD1 in maintaining and rescuing normal mitochondrial functions. 

### 3.4. APE1 Dysregulates Mitochondrial Functions in CD4 T Cells during HIV Infection 

**Figure 4:**
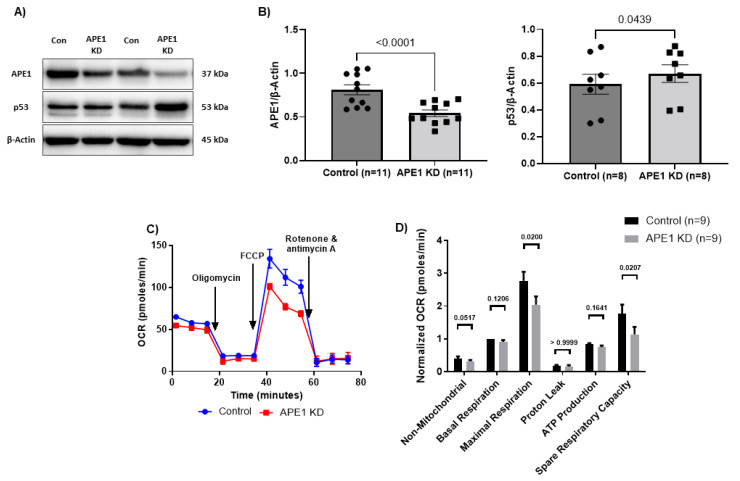
Mitochondrial functions following APE1 knockdown in CD4 T cells from HS. (**A**,**B**) Representative Western blots and summary data of APE1 (*n* = 11) and p53 (*n* = 8) protein levels following APE1 KD in CD4 T cells from HS normalized to β-Actin. (**C**,**D**) Oxygen consumption rate (OCR) as measured using Seahorse XFp Cell Mito Stress Tests, and summary data for non-mitochondrial, basal, and maximal respiration, proton leak, ATP production, and spare respiratory capacity following APE1 KD (*n* = 9). KD—knockdown.

APE1 is an endonuclease related to the BER pathway. To investigate the link between APE1 and ROS-mediated mtDNA damage and mitochondrial dysfunction, we knocked down APE1 in primary CD4 T cells from HS. We observed significantly reduced APE1 protein levels 72 h after the KD (Figure 4A,B). Since APE1 functions in both antioxidant metabolism and DNA damage repair, we investigated the effect of APE1-KD in HS-CD4 T cells on the level of oxidative stress, mtDNA copy number and mtDNA damage, cellular respiration, ATP production, and apoptosis. We observed no significant changes in cellular ROS (Supplementary Figure S3A), mitochondrial ROS (Supplementary Figure S3B), mitochondrial mass (Supplementary Figure S3C), mitochondrial membrane potential (Supplementary Figure S3D), mtDNA copy number (Supplementary Figure S3E), or percentage of mtDNA damage (Supplementary Figure S3F). As shown in Figure 4C,D, we did, however, observe significantly reduced maximal respiration and spare respiratory capacity following APE1-KD in HS-CD4 T cells, indicating the presence of impaired mitochondrial function. Recent evidence has shown that inhibition of APE1 enzymatic activity regulates mitochondrial respiration via a p53-dependent mechanism [61]. Thus, we assessed the effect of APE1-KD on p53 protein levels. We observed a significant increase in p53 protein level following APE1-KD (Figure 4B), suggesting the presence of a p53-mediated mechanism that regulates mitochondrial functions via downregulation of APE1 protein.

**Figure 5:**
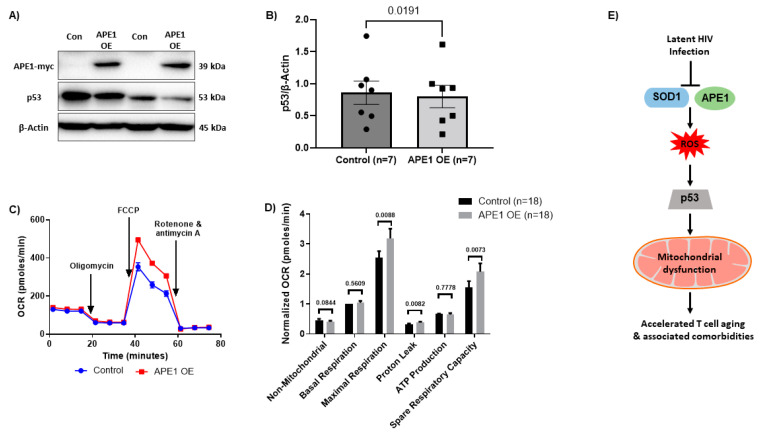
Mitochondrial functions following APE1 overexpression in CD4 T cells from PLWH and model of latent HIV infection induced mitochondrial dysfunction via multi-faceted synergistic mechanisms. (**A**,**B**) Representative Western blots and summary data of APE1-myc and p53 protein levels following APE1 OE in CD4 T cells from PLWH normalized to β-Actin (*n* = 7). (**C**,**D**) OCR and summary data for non-mitochondrial, basal, and maximal respiration, proton leak, ATP production, and spare respiratory capacity following APE1 OE (*n* = 18). OE—overexpression. (**E**) A model depicting the molecular mechanism linking deregulations of APE1 and SOD1 protein levels and mitochondrial compromise via influencing multiple pathways during latent HIV infection. These deregulations result in mitochondrial failure and reduced OXPHOS, as well as accelerated aging and CD4 T cell dysfunction during latent HIV infection.

To evaluate if restoring APE1 protein level would rescue HIV-CD4 T cell mitochondrial functions, we overexpressed APE1 in HIV-CD4 T cells. We assessed transfection efficiency by measuring the presence of the myc-APE1 tagged protein and observed successful APE1 overexpression following lentiviral transfection (Figure 5A). Similar to SOD1-OE, we did not find significant changes in cellular ROS (Supplementary Figure S3G), mitochondrial ROS (Supplementary Figure S3H), mitochondrial mass (Supplementary Figure S3I), mitochondrial membrane potential (Supplementary Figure S3J), or mtDNA copy number (Supplementary Figure S3K) following APE1-OE, but did observe a significant rescue of maximal respiration and spare respiratory capacity after APE1-OE (Figure 5C,D). We further evaluated if the rescue of mitochondrial functions may be mediated by restoring DNA damage-mediated activation of p53. Western blotting showed a significant reduction in p53 protein following APE1-OE (Figure 5B). These results suggest that mitochondrial cellular respirations were inhibited or rescued by APE1 KD in HS-CD4 T cells or OE in HIV-CD4 T cells, respectively, and that this process is likely mediated via p53 activation.

## 4. Discussion

We and others have shown evidence that latent HIV infection induces T cell oxidative stress, nuclear and telomeric DNA damage and failure to repair, and mitochondrial dysfunction, resulting in premature T cell aging in PLWH [15,16,17,19,23,26,28]. Mitochondrial functions are important for cellular responses, with essential roles in maintaining T cell activation, proliferation, survival, and effector functions, highlighting the relationship between immune function and cellular metabolism [62,63,64,65,66,67]. T lymphocytes exhibit high energetic demands to retain clonal expansion and effector functions, forcing T cells to maintain a highly active cellular metabolism, resulting in increased production of ROS. While ROS are vital second messengers in normal T cell activation and signaling, T cells are still susceptible to ROS-induced DNA damage and dysfunction [68].

mtDNA are particularly vulnerable to oxidative damage compared to nuclear DNA (nuDNA) due to the lack of histone protection and close proximity to ROS generation during OXPHOS [69]. Moreover, mtDNA damage has also been shown to persist longer than ROS-induced nuDNA damage [70]. It is widely accepted that alterations to the mitochondrial genome are always present; however, these alterations only affect mitochondrial or cellular function once they reach a threshold of around 50%, at which the ratio of mutated mtDNA compared to wildtype mtDNA is sufficient to influence the electron transport chain (ETC) [57,71]. Cellular respiration is facilitated by the components of the ETC, including four multimeric mitochondrial encoded enzymes (complexes I-IV), ATP synthase, and the electron carriers coenzyme Q (CoQ) and cytochrome *c* [72]. While mtDNA only encodes a portion of the necessary components (13 proteins) responsible for cellular respiration, the majority of the proteins responsible for mtDNA expression and maintenance, structural subunits for OXPHOS, factors required for assembly of mitochondrial OXPHOS machinery, and enzyme turnover are nuclear encoded. These nuclear-encoded gene products are transcribed, translated by cytoplasmic ribosomes, and translocated into mitochondria via membrane transporters, indicating a critical relationship between nuclear genome integrity, coordinate expression of mtDNA, and mitochondrial functions [72,73,74,75,76].

We have previously shown mitochondrial dysfunction, including reduced mtDNA content, elevated oxidative stress-induced DNA damage, and reduced mitochondrial respiration, in CD4 T cells from PLWH, particularly in individuals with incomplete immune reconstitution, referred to as immune non-responders (INRs) [28]. Surprisingly, we did not observe alterations in the mtDNA copy number or percentage of mtDNA damage after manipulation of SOD1 or APE1 in this study. However, we found that manipulation of SOD1 or APE1 was sufficient to significantly alter mitochondrial functions and cellular respiration likely via a p53-dependent mechanism. Notably, multiple copies of mtDNA, which is synthesized continually independent of cellular division, are dispersed within individual mitochondria. The number of mitochondria present in each cell varies based on the life stage of the cell, oxidative stress levels, and mitochondrial fusion, fission, and biogenesis [45,57,77,78,79,80,81]. Thus, it is possible that the experimental conditions used in the current study, representing acute deregulation, are not sufficient to alter mtDNA copy number to mimic the phenotypes observed in latent HIV infection, which represents chronic deregulation.

During normal DNA repair, oxidatively damaged DNA bases are recognized and cleaved by a DNA glycosylase, generating an abasic site (apurinic/apyrimidinic site, AP site). AP sites can then be recognized by APE1, an endonuclease able to cleave the AP site to generate a 3′-hydroxyl that can be further filled and ligated [36]. Oxidative stress oxidizes RNA 10–20 fold higher than DNA, leading to modification of RNA structure and function, suggesting that ROS-induced oxidation of RNA may be more severe and require mechanisms for repair [82,83,84,85]. DNA and RNA typically have different enzymes required for repair and dynamics; however, a few DNA repair proteins have been shown to be critical in regulating RNA quality control and removing damaged RNA that can ultimately lead to failed protein synthesis. Interestingly, APE1 has been shown to have enzymatic activity on RNA [86]. APE1 degrades dysfunctional abasic mRNA in mitochondria, whereby loss of APE1 expression in mitochondria results in accumulation of damaged mitochondrial mRNAs, impaired protein translation, and reduced mitochondrial encoded protein expression, directly influencing mitochondrial respiration [87]. Interestingly, studies have also shown that in response to increased oxidative stress, including APE1 KD, mitochondria can increase mtDNA content to reduce the accumulation of detrimental ROS-induced mtDNA damage and impairment of mitochondrial protein synthesis [87,88,89].

APE1 plays a role in regulating the DNA-binding activity (via changing the adjacent cysteine between disulfide bond and thiol groups) of nuclear NRF-1, which is a critical transcription factor for mitochondrial genes, including Mitochondrial transcription factor A (TFAM), Cytochrome C Oxidase Subunit 6C (COX6c), and Translocase Of Outer Mitochondrial Membrane 22 (TOMM22) [82]. These downstream nuclear-encoded genes are essential for mtDNA transcription and biogenesis, electron transport via the ETC, and translocation of nuclear-encoded proteins across the outer mitochondrial membrane, respectively. These findings could explain why we did not observe alterations in mtDNA content or mtDNA damage but did find changes in mitochondrial respiration following APE1 manipulation in the current study. It is possible that APE1 influences mitochondrial function not by directly protecting from oxidative stress or repairing mtDNA damage, but by ensuring the necessary components required for normal mitochondrial function are properly transcribed, translated, and translocated to mitochondria to carry out their normal functions.

SOD1 is considered to be the first line of defense against oxidative stress and is essential for oxygen and nutrient sensing. Cells have adaptive mechanisms to restore mitochondrial fitness and reduce cellular death via antioxidant systems, mitochondrial proteases and chaperones, and mitochondrial biogenesis and mitophagy; however, oxidative stress can damage DNA, lipids, and proteins, and leads to protein misfolding [37,90]. The mitochondrial unfolded protein response (UPR^mt^) is involved in the activation of multiple parallel transcription factors (TFs) and signaling cascades involved in ROS-mediated damage responses. Specifically, the UPR^mt^ is a retrograde signaling axis from the mitochondria to the nucleus via the TF C/EBP homologous protein (CHOP), resulting in the activation of mitochondrial chaperones and proteases. Studies have shown that SOD1 has a critical role in the activation of the UPR^mt^ and in protecting cells from mitochondrial oxidative stress and cell death. SOD1 has also been shown to associate with and promote the activity of ERRα, a nuclear receptor essential in regulating mitochondrial biogenesis, gluconeogenesis, oxidative phosphorylation, and fatty acid metabolism via the PGC-1α-mtTFA signaling pathway [91,92]. Together, these data imply that SOD1 acts in a multi-faceted approach to protect the mitochondria via antioxidant function, maintaining communication between the nucleus and mitochondria to signal for metabolic gene transcription, and protect the mitochondria from the accumulation of damage.

At this point, the exact mechanisms by which APE1 and SOD1 protect cellular respiration remain incompletely understood, however, our data suggest that APE1 and SOD1 may regulate mitochondrial functions by regulating p53 activation. We have previously shown that p53 is upregulated during chronic viral infection [19]. We have also demonstrated deregulations of the master mitochondrial regulators PGC-1α, ERRα, NRF-1, and mtTFA in CD4 T cells via a p53-dependent mechanism during telomere targeting and latent HIV infection [27,28]. These proteins are essential for normal mitochondrial biogenesis and mtDNA maintenance. Our data suggest that during HIV infection, the disturbances of critical antioxidant and DNA damage repair proteins (SOD1, and APE1) regulate p53 activation, which may further influence the levels of master mitochondrial regulators, leading to synergistic deregulation of mitochondrial function. In line with this, our data suggest that a multi-layer mechanism involving ROS production, mtDNA/nuDNA, p53 regulation, and UPR^mt^-activated TF activity maintains normal mitochondrial functions to ultimately facilitate T cell immunity. Based on our findings in this study and the published literature, we propose a model as shown in Figure 5E. We believe that HIV-induced deregulation of SOD1 and APE1 proteins creates a cascade of signals that ultimately contribute to mitochondrial failure, including elevated ROS that facilitate damage to both nuclear and mitochondrial DNA, RNA, and proteins, leading to p53-mediated inhibition of master mitochondrial regulators PGC-1α, mtTFA, NRF-1, and ERRα, accumulation of dysfunction abasic mRNAs that can lead to reduced mitochondrial proteins, and inhibition of the UPR^mt^. These synergistic and multi-leveled machineries become dysregulated during latent HIV infection, leading to increased oxidative stress, mitochondrial compromise, accelerated aging, reduced energetic output, CD4 T cell dysfunction, and immune failure. The inner workings of this complex mechanism warrant further investigations, which are underway in our laboratory. This study suggests that a combination of multi-target approaches may be necessary to repair mitochondrial functions, and the diminished immune responses and accelerated CD4 T cell aging during latent HIV infection.

## Figures and Tables

**Table 1:** Characteristics of the study subjects.

Subjects	*n*	Sex	Median Age	Median CD4 Count (Cells/μL)
HS	46	37M/9F	42 (21–70)	N/A
HIV	77	67M/10F	53 (19–78)	719 (85–2223)

## Data Availability

Data available on request.

## Supplementary Materials

The following supporting information can be downloaded at: https://www.mdpi.com/article/10.3390/v15051061/s1, Figure S1: Mitochondrial readouts following SOD1 manipulation in CD4 T cells; Figure S2: Mitochondrial readouts following PRDX1 knockdown in HS-CD4 T cells; Figure S3: Mitochondrial readouts following APE1 manipulation in CD4 T cells.
