# Supplementary material for: ROS-Induced Mitochondrial Dysfunction in CD4 T Cells from ART-Controlled People Living with HIV
Source: Viruses. 2023 Apr 26;15(5):1061. doi: 10.3390/v15051061 (PMC10224005; doi:10.3390/v15051061)
Supplement: Supplementary file 1 [file viruses-15-01061-s001.zip › viruses-2313489-supplementary.pdf]

Supplemental Figures

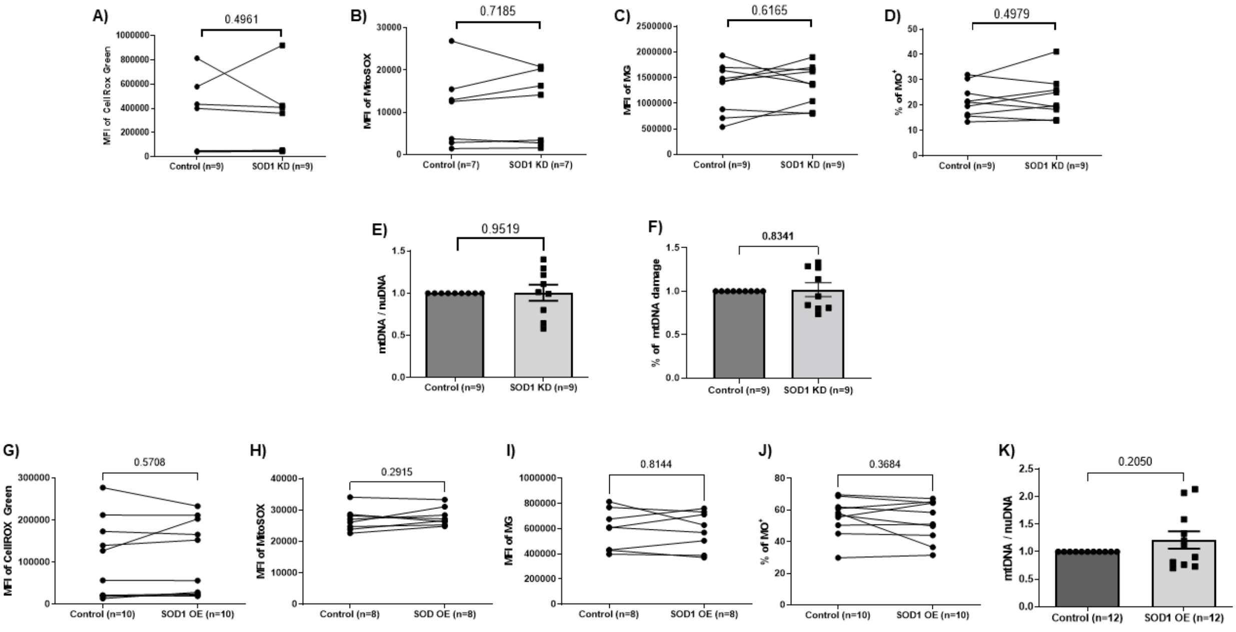

Supplemental Figure S1. Mitochondrial readouts following SOD1 manipulation in CD4 T cells. (A) MFI of CellROX Green following SOD1 KD (n=9). **(B)** MFI of MitoSOX following SOD1 KD (n=7). **(C-D)** MFI of MG (mitochondrial mass) (**C**) and percentage of MO^+ (**D**) following SOD1 KD (n=9). **(E-F)** Mitochondrial DNA (mtDNA) relative to nuclear DNA (nuDNA) content and percentage (%) of mtDNA damage, analyzed by qPCR following SOD1 KD (n=9). **(G)** MFI of CellROX Green following SOD1 OE (n=10). (**H)** MFI of MitoSOX following SOD1 KD (n=8). **(I-J)** MFI of MG (mitochondrial mass) (*I*, n=8) and percentage of MO^+ (*J*, n=10) following SOD1 OE. **(K)** mtDNA relative to nuDNA content, analyzed by qPCR following SOD1 OE (n=12). KD, knockdown; OE, overexpression.

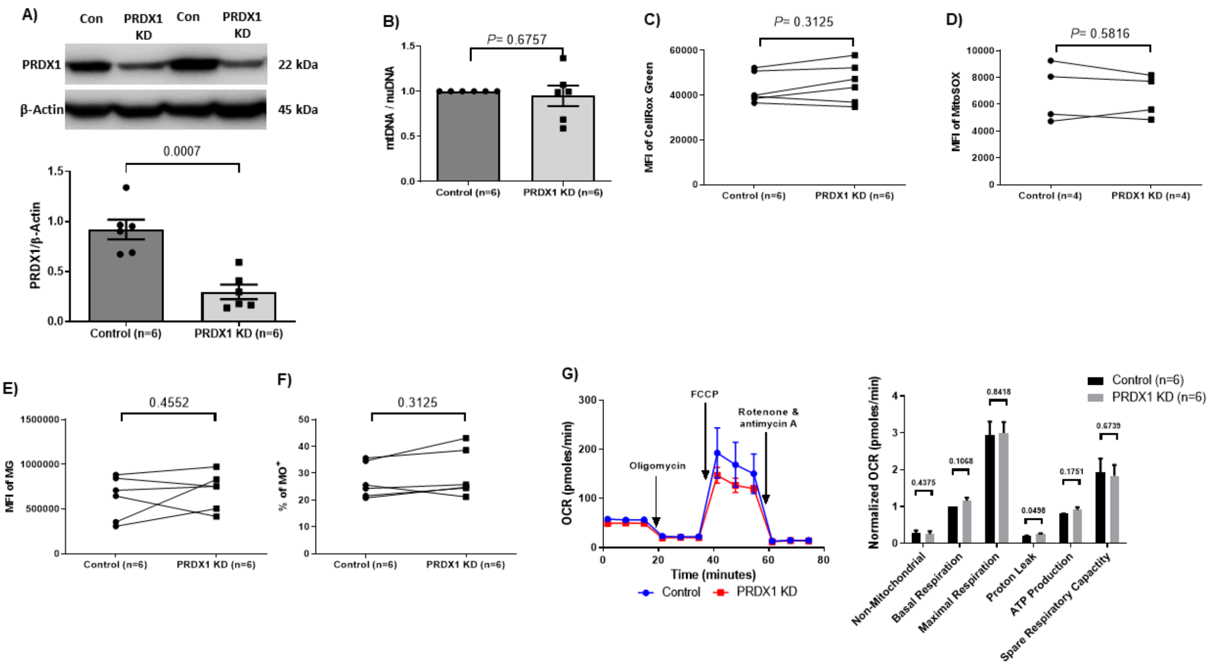

Supplemental Figure S2. **Mitochondrial readouts following PRDX1 knockdown in HS-CD4 T cells. (A)** Representative western blots and summary data following CRISPR/Cas9 mediated KD of PRDX1 in HS-CD4 T cells. **(B)** mtDNA copy number relative to nuclear DNA following PRDX1 KD in HS-CD4 T cells (n=6). **(C)** MFI of CellROX Green following PRDX1 KD (n=6). **(D)** MFI of MitoSOX following PRDX11 KD (n=4). **(E-F)** MFI of MG (mitochondrial mass) (*E*) and percentage of MO^{+} (*F*) following PRDX11 KD (n=6). **(G)** The OCR and summary data for non-mitochondrial, basal, and maximal respiration, proton leak, ATP production, and spare respiratory capacity following PRDX1 KD (n=6). KD, knockdown.

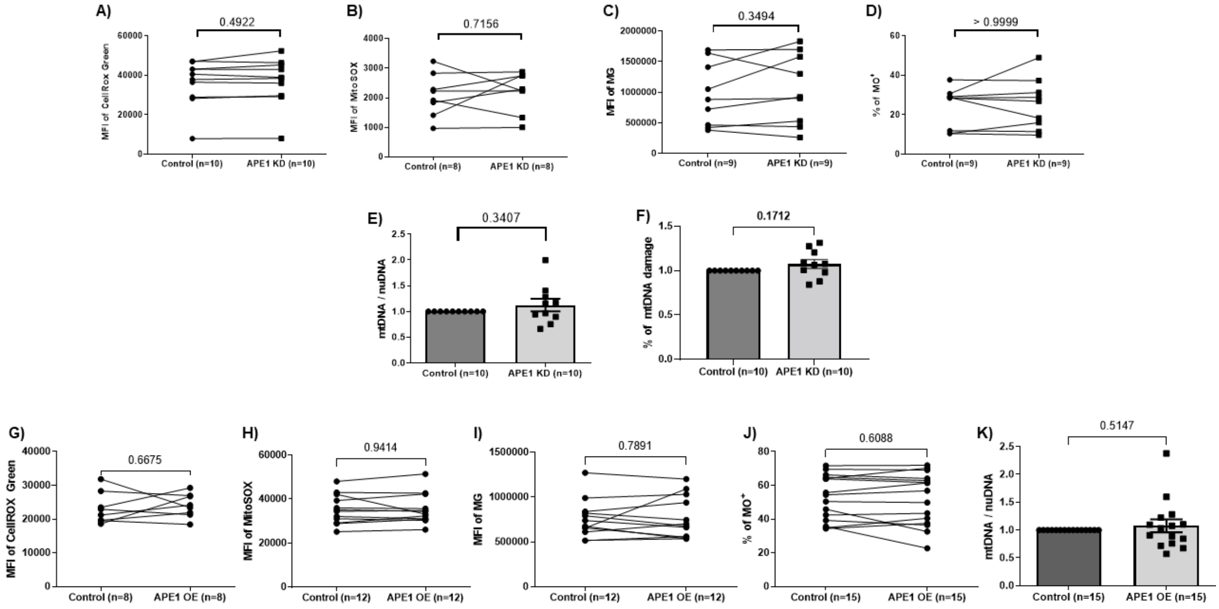

Supplemental Figure S3. Mitochondrial readouts following APE1 manipulation in CD4 T cells. (A) MFI of CellROX Green following APE1 KD (n=10). **(B)** MFI of MitoSOX following APE1 KD (n=8). **(C-D)** MFI of MG (mitochondrial mass) (*C*) and percentage of MO^+ (*D*) following APE1 KD (n=9). **(E-F)** Mitochondrial DNA (mtDNA) relative to nuclear DNA (nuDNA) content and percentage (%) of mtDNA damage following APE1 KD, analyzed by qPCR (n=10). **(G)** MFI of CellROX Green following APE1 OE (n=8). **(H)** MFI of MitoSOX following SOD1 KD (n=12). **(I-J)** MFI of MG (mitochondrial mass) (*I*, n=12) and percentage of MO^+ (*J*, n=15) following APE1 OE. **(K)** mtDNA relative to nuDNA content following APE1 OE, analyzed by qPCR (n=15). KD, knockdown; OE, overexpression.
